# Application of the age-period-cohort model in tuberculosis

**DOI:** 10.3389/fpubh.2025.1486946

**Published:** 2025-01-29

**Authors:** Dan Luo, Fengying Wang, Songhua Chen, Yu Zhang, Wei Wang, Qian Wu, Yuxiao Ling, Yiqing Zhou, Yang Li, Kui Liu, Bin Chen

**Affiliations:** ^1^School of Public Health, Hangzhou Medical College, Hangzhou, China; ^2^Department of Tuberculosis and AIDS Control and Prevention, Jinhua Municipal Center for Disease Control and Prevention, Jinhua, China; ^3^Department of Tuberculosis Control and Prevention, Zhejiang Provincial Center for Disease Control and Prevention, Hangzhou, China; ^4^School of Public Health, Health Science Center, Ningbo University, Ningbo, China; ^5^School of Public Health, Hangzhou Normal University, Hangzhou, China

**Keywords:** tuberculosis, age-period-cohort models, time trends, model application, identification problem

## Abstract

Up to now, tuberculosis (TB) remains a global public health problem, posing a serious threat to human health. Traditional methods for analyzing time-varying trends, such as age and period, tend to ignore the poor impact of birth cohorts, which is an important factor in the development of TB. The age-period-cohort (APC) model, a statistical method widely used in recent decades in economics, sociology, and epidemiology, can quantitatively estimate the efficacy of different age, period, and birth cohort groups for TB by separating the effects of these three dimensions and controlling for confounding factors among the time variables. The purpose of this paper is to briefly review the model, focus on the application of the existing APC model in the field of TB, and explain its advantages and disadvantages. This study will help to provides a theoretical basis and reference for using the APC model in TB analysis and prediction.

## Introduction

According to the Global Tuberculosis Report 2023, tuberculosis (TB) is the second leading cause of death worldwide from a single source of infection, with an estimated 10.6 million TB cases by 2022. TB remains a serious public health challenge (1). In addition to traditional risk factors, such as age, sex, comorbidities like HIV or diabetes, and malnutrition, period and birth cohorts have also been found to be associated with TB-related incidence (2–6). In 1939, Frost first isolated three time-varying factors affecting the disease in TB research: age, period, and birth cohort (7). Over the years, the concept and implementation method of age-period-cohort (APC) model has gradually matured and improved, and have been widely used in fields such as epidemiology and sociology (8). In TB research, APC modeling analyzes TB dynamics cross-sectionally and longitudinally by examining morbidity and mortality in populations of various regions at different ages, during different periods, and in birth cohorts. Due to differences in individual conditions at various ages, such as physical development in youth and physiological decline in older age; environmental changes at specific periods, such as the improvement of the healthcare system or the implementation of public health policies; and differences in birth cohort exposure to certain epidemics, the APC model can quantitatively assess the impact of age, period, and cohort factors on TB in different regions. This allows for more accurate identification, interpretation, and prediction of time trends and development patterns related to TB (9, 10). Although the model suffers from unidentifiability problems due to covariance between variables, it can provide a unique perspective for understanding the long-term trend of TB and offers basic information for long-term public health surveillance as the model is gradually optimized (8, 11). In the fields of other infectious diseases, such as influenza, HIV/AIDS, and measles (12–15), the APC model has been widely applied to identify the risk factor in different age groups. However, the potential function of the APC model in tuberculosis has not yet been fully summarized. In this study, we searched databases such as Web of Science, Pubmed, and CNKI for tuberculosis-related studies based on age-period-cohort models with the end time of 19 February 2024. The purpose of this article is to provide a brief overview of the APC model and summarizes its application, development, and limitations in the field of TB.

## Overview of the APC model

### Concepts and methods

The APC model is a statistical analysis tool used to reveal and understand potential effects across periods and ages (10, 16). Age, period, and birth cohort are all time-varying factors in a broadest sense, and although they are expressed differently, they collectively contribute to the occurrence of fluctuating phenomena such as epidemicsoutbreaks. The purpose of fitting the APC model is to explore and accurately define the independent impact of various types of time-varying factors on the incidence of events (17, 18). In this model, “age” refers to the biological age, which representing different life stages and physiological states of individuals or groups, such as changes in physical health, income level, and exposure risk from adolescence to old age (9, 19). “Period” refers to the time of study or observation, and the main manifestation of this variable is to reflect the situation or general trend at a given time, such as an infectious disease pandemic, famine, economic crisis, changes in healthcare policies, and the launch of specific drugs (20). “Cohort” reflects the variation among people born in different years, representing the intersection of individual early experiences and the macro-social environment (21, 22). These factors enable the analysis and interpretation of the patterns, trends, and possible effects of the phenomenon under study.

The classical APC model assumes an additive relationship between the dependent variable and “age, period, and cohort,” with these three as independent variables and the “incidence of a phenomenon” in a certain period of time or population as the dependent variable. This relationship is assumed to follow a generalized linear model (GLM) with a certain probability distribution (10, 18). The general form of the model can be expressed as:


(1) $$Y_{ij}=\ln{\hat{R\;}}_{ij}=\ln\frac{{\hat{O\;}}_{ij}}{N_{ij}}=\mu +\alpha_{i}+\beta_{j}+\gamma_{k}+\varepsilon_{ij}$$

In Equation 1, i ( i =1,2, … …,I) ,j ( j =1,2, … …,J), and k ( k =1,2, …,K) represent age, period, and cohort variables, respectively. $Y_{ij}$ is the model-dependent variable that represents the logarithmic morbidity or mortality rate of the population in age group i during period j . ${\hat{R\;}}_{ij}$ is the expectation of the morbidity or mortality rate $R_{ij}$ for the population of age group i during period j . Assuming followed a Poisson distribution, ${\hat{O\;}}_{ij}$ is the expectation of the number of morbidities or deaths $O_{ij}$ during period j for the population in age group i and $N_{ij}$ is the number of exposed person-years in period j for the population in age group i . μ is the model intercept, which represents the logarithmic morbidity or mortality rate for the baseline age, period, and cohort group. $\alpha_{i}$ , $\beta_{j}$ , and $\gamma_{k}$ are the effects of age group i , period j , and cohort k , respectively. In this formula, $\varepsilon_{ij}$ represents the random error term, accounting for unexplained variance in the model (18). In the result parameter, the RR (relative risk) value is the ratio of age-specific rates in group p relative to reference group p0. The net drift value refers to the APC analogue of the estimated annual percentage change (EAPC) in the age-standardized rate (ASR), and the local drift represents the estimated annual percentage change over time specific to age group i (10, 23).

The core idea of the APC model is to quantify the values of parameter changes in the incidence of target events from one age group to the next, from one period to another, and from one birth cohort to the next. This involves fitting the regression relationship between the target event incidence rate and the three factors of age, period, and birth cohort. So as to explore their independent effects of the three dimensions of age, period and birth cohort on the target event (24).

### Advantages

When analyzing the incidence or trend of a certain phenomenon or event, traditional temporal trend research tends to highlights the limitations that may result from time-varying factors closely related to age in the data. It can be challenging to control for or eliminate overlapping relationships between age, period, and birth cohort, resulting in distorted trends in age or period (16). The APC model has unique advantages compared to traditional epidemiological methods like descriptive temporal trend analysis, cross-sectional studies, and longitudinal studies. Its better controls for confounding between variables and quantitatively estimates the phenomenon of groups of different ages, periods, and birth cohorts. Therefore, the APC model has been widely used by economists, sociologists, and public health researchers to address important issues, such as life-cycle income patterns, wage dynamics, social change, fertility rate, population aging, and the causes and incidence of life-threatening diseases (18, 25).

## Age, period, and cohort effects in tuberculosis

### Age effect

Age is globally recognized as one of the most significant risk factors for TB, and the age effect on TB, as determined by the APC model, varies widely between countries and regions (26, 27). Detailed information on the available studies is shown in Supplementary Table S1-1.

Age effects in some areas have shown that children aged 0–14 years are a special population for TB. For instance, data from 1996 to 2016 on TB incidence in the United States and from 1961 to 1990 on TB mortality rate in Taiwan, China, showed that this age group was at high risk (28, 29). As population of children enter public places, such as schools, the relative concentration of people increases their risk of exposure (30). However, this age group was considered low-risk in studies based on TB mortality in the United States from 1900 to 1950 (28).

Individuals aged 15–64 years are also a high-risk group in some countries and regions, such as some developed countries (United States, Russia, Japan, Netherlands, Italy, New Zealand) and countries in Africa such as Cameroon (CAM), Central African Republic (CAR), Chad, and the Democratic Republic of the Congo (DRC). Meanwhile, risk effects within this age group have also been observed in morbidity and mortality studies in 204 countries and territories worldwide and some regions of China (2, 4, 9, 25, 28, 31–42).

In the vast majority of countries and regions, APC for TB-related studies indicates risk effects in people over 65 years old, including mortality from alcohol consumption in 204 countries and territories worldwide, as well as morbidity and mortality in the United States, India, South Africa, and some regions of China (9, 29, 31, 33, 35, 38, 39, 41–43). Older people are at a higher risk of morbidity and mortality than other age groups due to their age, decreased immune and cognitive performance, inadequate social and family support, poor nutritional status, and various comorbidities (5, 6).

The tuberculosis burden was varied across nearly all age groups in distinct area and countries, which may be attributed to the difference in socioeconomic background and environmental deviation, dynamic changes in immune level of the whole group, and the historical context of local epidemics as well. Thus, it is hard to generalize the study results to other areas or contexts. For example, some regions showed a higher risk in young group like “0–14” age group is generally paralleled with poor public health conditions, low BCG coverage and malnutrition (28, 29). In addition, the older adults aged 65 and above was being the focus of tuberculosis control in developing countries, which was due to the high historical epidemic levels and ageing along with malnutrition and chronic diseases (5, 6).

### Period effect

Period-effect studies on TB date back to the mid-20th century and extend to 2020. Overall, the period effect of TB-related morbidity and mortality has been declining globally. Three studies based on 204 countries and territories around the world show an overall downward trend in TB incidence and mortality due to tobacco, alcohol, or high fasting plasma glucose (HFPG) between 1990 and 2019 (2–4). Additionally, regional studies have shown that most countries, including developed countries (the United States, several European countries, Japan, South Korea, etc.) and developing countries (such as India, Cameroon, Central Africa, China, etc.) have shown a similar downward trend over time (9, 21, 25, 28, 30, 32, 34, 35, 37–48). Notably, some studies reported a brief increase over a specific period, followed by a steady decline. A study in the United States showed a rebound in the reported TB incidence from 1986 to 1992 compared to the total study period of 1953–2000 (49). Similarly, a study in China found a brief increase in the period of 2004 to 2005, followed by a downward trend (35). In addition, studies conducted in the United States, Japan, and the Netherlands showed an upward trend in TB mortality during the Spanish influenza epidemic (1918–1919), followed by a sharp decline (36). In summary, since the 20th century, the global TB epidemic situation has been stabilizing and decreasing with the intensification of TB prevention and control.

The period effect mainly reflects changes in tuberculosis trends across different historical periods. The temporary increases or decreases in period effects observed in different countries and regions are often closely related to public health policies at the time, economic crises, advancements in medical technology (such as the widespread use of anti-tuberculosis drugs), pandemics, drug resistance issues, vaccination coverage, and other public health developments. Major events in different periods have a profound impact on the disease burden and provide unique perspectives for evaluation and future control efforts.

### Cohort effect

Cohort effects based on APC models showed a decreasing trend in TB incidence or mortality studies due to high fasting glucose levels in 204 countries and territories. The highest cohort risk effect in TB mortality due to smoking and alcohol consumption was observed during 1990–1940 and 1990–1920, respectively (2–4). The global cohort effect of TB incidence or mortality on a country or territory basis has shown a downward trend, including Brazil, Russia, India, China and South Africa (BRICS); England and Wales, Italy, New Zealand, CAM, CAR, DRC, the Netherlands, Japan, and China (9, 30–38, 45–47). Among these, a Japanese study found that birth cohort effects were higher in 1913 and 1963 than in neighboring cohorts (37).

Additionally, several studies in the United States have shown a decline in the birth cohort effect on TB (28, 33, 36, 49). In contrast, a study showed a U-shaped birth cohort effect on TB incidence in the United States, peaking in 1992 and 2017, respectively (33).

It is worth noting that while many national-level birth cohort effects show a general downward trend, there are some specific birth cohorts within, such as China. A TB incidence study across China found that birth cohorts from 1961 to 1965 and 2001 to 2005 were at higher risk than adjacent birth cohorts (9). Another study from Fudan University showed that the cohort effect of TB morbidity and mortality was no longer a risk factor for individuals born after 1978 (38). The results of TB morbidity and mortality studies at the provincial level in China include high-risk cohorts in the Yunnan Province for 1962–1970 and 2001–2010, Taiwan for 1891–1921, Hong Kong for 1906, and Jiangsu Province for 1940–1950 for males and 1990 for females (21, 39, 40, 48). Although these effects were slightly different in birth cohorts that were higher than in neighboring cohorts, they all showed an overall downward trend, consistent with the trend on a global scale.

The cohort effect reflects the impact of the intersection between the early experiences of individuals born in different time periods and the broader social environment on tuberculosis. It is likely closely related to changes in the health status, lifestyle, tuberculosis exposure risks, treatment, and socioeconomic background of specific populations throughout their lives. Different historical exposures and intergenerationally identified high-risk cohorts can be used to anticipate future tuberculosis challenges through, for example, the impact of persistence.

## Application limitations and model optimization

Theoretically, the APC model can provide independent decomposition effects of the three dimensions of age, period, and birth cohort. However, in practice, there is a complete collinearity relationship between these factors expressed as “period−age = birth cohort,” leading the system to un-identifiability. Additionally, there is uncertainty regarding how to explain the parameter bias (10, 23). Traditional regression models are unable to obtain a unique solution for the model parameters, and this problem has been emphasized and discussed since 1973. Researchers have proposed various methods to address this issue, including two-factor models, constrained generalized linear models (CGLIM), nonlinear models, estimate function methods, penalty function approaches, individual records approaches, autoregressive age-period-cohort models, intrinsic estimators (IE), smoothing cohort models, sequential methods, and canonical parameterization approaches (17, 18, 23, 50, 51). Additionally, there is an overlap between birth cohorts calculated by grouping in the APC model, and most studies ignore this overlap by taking the median value of the birth cohort grouping (9, 52). In conclusion, with the wide application of the APC model in epidemiology, economics, and other fields, problems such as “identification” have been gradually addressed and optimized.

Currently, the most dominant APC modeling approaches in TB-related research are the estimable function and the IE approaches. The estimable function method proposed by Holford in 1983 estimates the model parameters by calculating the bias and curvature (52). In 2014, the US National Cancer Institute implemented estimable functions and Wald’s test in R. The sham design data followed a Poisson distribution, and statistical analyses were performed using the weighted least-squares method. They developed an easily accessible web tool for this method, and Shareen A. Iqbal applied it to tuberculosis research for the first time in 2018 (23, 25). The IE method based on estimating functions and matrix singular value decomposition was proposed by Fu in 2000 despite controversy due to the problem that the actual significance of the parameter estimates is not intuitive. However, the IE method is effective at estimating the independent effects of age, period, and cohort groups and has been used to evaluate the age, period, and birth cohort effects of TB morbidity and mortality (17, 53). Based on the findings from APC, more intensive tuberculosis interventions like supplementary vaccination and regular screening could be strengthened for specific age groups and birth cohort with high risk. Moreover, In the future, it will still be necessary to explore new perspectives and mechanisms to deepen the application of APC.

## Conclusion

The APC model decomposes age, period, and cohort effects by analyzing data from different age groups, periods, and birth cohorts. This approach provides insights into the characteristics of TB epidemics and develops appropriate preventive and control measures. With continuous optimization and improvement of statistical methods, as well as addressing “identification” problems, the application prospects of the APC model in the field of TB are more promising. In the future, establishing an APC model and continuously updating data will allow for the prediction and trend analysis of TB morbidity and mortality. Determining the effects of different age groups, periods, and birth cohorts will help identify relevant risk factors for TB and monitor trends over time. In addition, the results of the model can be used to assess the effectiveness of interventions and inform policy development. It is worth noting that, as a statistical analysis tool, the results of the APC model can be used as a reference for TB prevention and control rather than as a standard. The result of the analysis should be combined with the actual situation of the region and multifaceted research and judgment, followed by the adjustment of policy priorities, resource allocation, and the implementation of targeted interventions.
