# Supplementary material for: Application of the age-period-cohort model in tuberculosis
Source: Front Public Health. 2025 Jan 29;13:1486946. doi: 10.3389/fpubh.2025.1486946 (PMC11814438; doi:10.3389/fpubh.2025.1486946)
Supplement: Supplementary file 1 [file Table_1.DOCX]

Table S1-1 Application list of the age-period-cohort model in tuberculosis.

| **ID** | **Author** | **Study area** | **TB indicators** | **Study period** | **Statistical method** | **Note** |
| --- | --- | --- | --- | --- | --- | --- |
| 1 | Zou Z et al. | BRICS | Mortality | 1990-2019 | Estimate Function |  |
| 2 | Iqbal SA et al. | United States | Incidence | 1996–2016 | Estimate Function |  |
| 3 | Noymer A et al. | United States | Mortality | 1990-1950 | Others |  |
| 4 | Courval JM et al. | United States | Incidence | 1953-2000 | Other | An epidemiology Abstracts |
| 5 | Wu J et al. | 204 countries and territories | Age-standardized death rates attributed to alcohol and cigarette | 1990-2019 | Estimate Function |  |
| 6 | Wang L et al. | 204 countries and territories | Age-standardized prevalence rate of HIV and DS-TB co-infection | 1990-2019 | Others | APC model for prediction |
| 7 | Lan Q et al. | 204 countries and territories | Incidence | 1990-2019 | Estimate Function |  |
| 8 | Wang C et al. | 204 countries and territories | HFPG-related TB mortality | 1990-2019 | Estimate Function |  |
| 9 | Cui Y et al. | United States, China and India | Incidence | 1992-2017 | Intrinsic Estimator |  |
| 10 | Martial NT et al. | CAM, CAR, DRC | Incidence and mortality | 1990-2019 | Estimate Function |  |
| 11 | Yun JW et al. | South Korea | Mortality | 1983-2012 | Others | APC model for prediction |
| 12 | Collins JJ et al. | Italy, England and Wales, and New Zealand | Mortality | 1861-1971 (England and Wales), and 1881-1971 (Italy and New Zealand) | Others |  |
| 13 | Dhamnetiya D et al. | India | Incidence and mortality | 1990-2019 | Estimate Function |  |
| 14 | Oei W et al. | United States, Japan, and the Netherlands | Mortality | 1900-1940 (United States), 1899-1943 (Japan), and 1901-1940 (Netherlands) | Others |  |
| 15 | Ota M et al. | Japan | Incidence | 1953-2022 | Intrinsic Estimator |  |
| 16 | Dong Z et al. | China | Incidence | 2006-2020 | Intrinsic Estimator |  |
| 17 | Wei X et al. | China | Incidence | 2004-2018 | Intrinsic Estimator |  |
| 18 | Wang L et al. | China | Incidence and mortality | 2004-2019 | Intrinsic Estimator |  |
| 19 | Li S et al. | China | Incidence and mortality | 1990-2019 | Estimate Function |  |
| 20 | Haritebieke S et al. | China | Incidence | 1990-2019 | Intrinsic Estimator |  |
| 21 | Jiang Y et al. | China | Incidence | 1990-2019 | Intrinsic Estimator |  |
| 22 | Liu S et al. | China | Incidence in 0-10 years old | 2004-2018 | Estimate Function |  |
| 23 | Lin Q et al. | Shandong, China | Incidence | 2004-2017 | Canonical parameterization | APC model for prediction |
| 24 | Li L et al. | Taiwan, China | Mortality | 1961-1990 | Others |  |
| 25 | Zhang Y et al. | Jiangsu, China | Incidence and mortality | 2005-2020 | Estimate Function |  |
| 26 | Ji W et al. | Jiangsu, China | Incidence | 2009-2018 | Canonical parameterization |  |
| 27 | Cheng Q et al. | Sichuan, China | Incidence | 2005-2018 | Intrinsic Estimator |  |
| 28 | Wu P et al. | Hong Kong, China | Incidence | 1961-2005 | Others |  |
| 29 | Chen J et al. | Yunnan, China | Incidence | 2006-2020 | Estimate Function |  |

Connect to the table above:

| **ID** | **Age effects** | **Period effects** | **Cohort effects** |
| --- | --- | --- | --- |
| 1 | India and South Africa are rising, Russia is 45-49 years old, and China is decline | China, Brazil, and India are decline, Russia and South Africa are up in the first 10 years and down in the last 15 years | Decline |
| 2 | 0-5 and 20-30 years old | Decline | Decline |
| 3 | Highest for ages 20-35, lowest for ages 5-14 | Decline | Decline |
| 4 | Rising | Resurgence in 1986-1992 | Decline |
| 5 | 60-89 for mortality due to alcohol consumption, 40-60 for mortality due to smoking | Decline | 1990-1920 for mortality due to alcohol consumption, 1990-1940 for mortality due to smoking |
| 6 | — | — |  |
| 7 | 23 years old | Decline | Decline |
| 8 | 55-59 | Decline | Decline |
| 9 | 20-35 and 60-65 in the United States, 75-79 in China and India | China and India are declining, the United States in 1997 | India is down, China is 1942, and the United States has declining risk until 1957 and rising after 1957 |
| 10 | Incidence and mortality are 15-54 and 15-49, respectively | Decrease in incidence and mortality | Decrease in incidence and mortality |
| 11 | Rising | Decline | — |
| 12 | Declines after birth, peaks in 20-30 years old | Italy, England and Wales was flat and unchanged until 1951, and declined sharply after 1956, and New Zealand was 1956 | Overall decline |
| 13 | Rising | Decline | Decline |
| 14 | 15-25 years old | Rise in 1918-1919, followed by sharp decline | W-shaped fluctuation down |
| 15 | 20-29 years old | Late 1950s and early 1960s | 1913 and 1963 were higher than neighboring cohorts and declined overall |
| 16 | 20-24 and 70-74 years old | Decline | Slightly higher in 1961-1965 and 2001-2005, and declining overall |
| 17 | 25-29 and 70-74 years old | Slightly higher in 2005-2006, with an overall decrease | Decline |
| 18 | 20-24 and 70-74 for incidence, and persons over 60 years for mortality | Incidence declined and mortality stabilized | Overall decline and not a risk factor after 1978 |
| 19 | 20-24 for the whole population and women, and for males the morbidity and mortality rates are 65-69 and 30-34, respectively | Decline | Decline |
| 20 | Rising | Decline | Decline |
| 21 | Rising, with peaks at ages 90-94 | Decline | Decline |
| 22 | Decreasing from 0-3 years, and increasing from 3-10 years | Decline | Decline |
| 23 | — | — | — |
| 24 | 0-4 and 70-74 years old | Decline | 1891-1921 |
| 25 | 20-24 and 70-74 for incidence, and 80-84 for mortality | Decline | Decline |
| 26 | 20-30 and 60-80 years old | Decline | 1940-1950 for male, and 1990 for female and external population |
| 27 | — | — | Birth cohort experiencing famine, with intergenerational effects in this population |
| 28 | 20-24 years old | Overall U-shape and began to rise in 1971 | Rising before 1906 and falling after 1906 |
| 29 | 65-69 and 25-29 years old | Decline | Higher than neighboring cohorts in 1961-1970 and 2001-2010, with an overall downward trend |
